# Effects of AD3EC and Ca‐P‐Mg Supplementation on Oxidative Stress in Postpartum Dairy Cows

**DOI:** 10.1155/vmi/9584621

**Published:** 2026-07-21

**Authors:** Ameneh Khoshvaghti, Farhad Boti, Saeed Zahmatkesh, Mohaddeseh Shahriyari

**Affiliations:** ^1^ Department of Clinical Sciences, Kaz.C., Islamic Azad University, Kazerun, Iran, azad.ac.ir

**Keywords:** dairy cow, dry period, mineral supplement, oxidative stress, vitamin supplement

## Abstract

In industrial cattle breeding, special attention is paid to the nutrition of dairy cow livestock production while maintaining animal health. Vitamins and trace minerals such as zinc, copper, and manganese are essential for the functioning of many important proteins in the body. The body’s antioxidant system plays a critical role in cell health and, consequently, in the health of body tissues and organs. This defense system helps maintain tissue health by protecting cells against free radicals. Oxidative stress has been found to play a role in the occurrence of many diseases, including metabolic and infectious diseases in organisms. This study measured the effect of vitamin and mineral supplements on serum enzyme antioxidant levels and malondialdehyde concentrations in postpartum dairy cows. In this research, 15 cows received a subcutaneous vitamin supplement (AD3EC) on Day 210 of gestation (single dose) and a single subcutaneous dose of calcium, phosphorus, and magnesium immediately after calving (as a method to enhance the antioxidant immune system of dairy cows due to antioxidant effects of vitamins and minerals, particularly magnesium). These cows were compared to 15 cows that did not receive any vitamin supplements during the dry period but received only calcium in the form of an oral bolus immediately after calving. Serum enzyme antioxidant levels and malondialdehyde were measured using commercial laboratory kits according to standard methods. The results showed the MDA levels decreased but catalase and TAC levels increased in the group that used vitamin and mineral supplements. However, there was no significant change in other parameters. In conclusion, while the consumption of mineral and vitamin supplements does not significantly alter the mean concentrations of superoxide dismutase and glutathione peroxidase, it can reduce oxidative stress by improving the other oxidative stress parameters in dairy Holstein cows.

## 1. Introduction

In industrial dairy farming systems, considerable emphasis is placed on the formulation of precise dietary regimens for cattle, aiming to maximize livestock productivity while concurrently preserving animal health. The presence of essential vitamins and trace minerals, including zinc, copper, manganese, and others, is fundamental for the optimal functionality of numerous critical proteins within the organism [1]. However, due to the intensified production demands inherent in industrial husbandry practices and the potential insufficiency of these micronutrients in the dietary composition, the incidence of various diseases is likely to increase [2]. The body’s antioxidant defense system, encompassing both enzymatic and nonenzymatic antioxidants, plays an indispensable role in maintaining cellular integrity and, consequently, the overall health of tissues and vital organs [2].

Enzymes such as superoxide dismutase (SOD), glutathione peroxidase (GPX), and catalase, along with specific vitamins and proteins including vitamin E, carotenoids, and vitamin C, play integral roles within this system. Impairment in the functionality of this antioxidant defense system may predispose the organism to the development of various diseases.

GPX functions as an enzymatic antioxidant defense mechanism involved in a wide range of physiological processes. This enzyme catalyzes the reduction of hydroperoxides, including hydrogen peroxide, by utilizing reduced glutathione (GSH), thereby protecting the cell against oxidative damage. Except for phospholipid‐hydroperoxide GPX, which exists as a monomer, all GPX enzymes are tetramers composed of four identical subunits. Each subunit contains a selenocysteine residue at its active site, which directly participates in the two‐electron reduction of peroxide substrates.

Catalase is a heme‐containing enzyme localized within cellular peroxisomes, playing a critical role in detoxification by decomposing hydrogen peroxide (H_2_O_2_) into water and molecular oxygen. Hydrogen peroxide is generated as a by‐product of various cellular reactions, and its accumulation can lead to oxidative damage. Consequently, catalase serves to protect cells against oxidative injury.

Malondialdehyde (MDA) is one of the key biomarkers of lipid peroxidation in cells and tissues, produced as a result of lipid oxidation processes.

The antioxidant defense system contributes to the maintenance of tissue health by protecting cells against free radicals. In recent years, numerous studies have demonstrated that oxidative stress plays a significant role in the pathogenesis of various diseases, including metabolic and infectious disorders in animals. Therefore, it is suggested that enhancing the body’s antioxidant system may improve resistance to various diseases. This is particularly crucial in animals such as dairy cattle, where maintaining health has a profound impact on production parameters, including milk yield, meat quality, and reproductive performance [2]. Accordingly, it is essential to consider strengthening the antioxidant defense mechanisms during the dry period and immediately postpartum, alongside other management practices. Nevertheless, in industrial dairy farms, there is still no standardized protocol for the supplementation of vitamins and minerals. In many operations, particularly regarding vitamin supplementation, no routine administration is implemented, while in others, the inclusion of certain vitamins is incorporated into routine management protocols.

In a study conducted by Mousavi et al. (2019), the effects of administering a solution containing vitamin E and selenium, as well as a solution comprising vitamin B12 and iron, were evaluated in pregnant dairy cows during the transition period, focusing on colostrum quality and the antioxidant capacity of calf serum. The researchers concluded that the injection of these solutions on Days 7 and 21 prior to parturition had no significant effect on the immune system of the calves within the first 24 h of life [3].

It has previously been reported that the number of parturitions does not have a significant effect on the incidence of subclinical hypocalcemia before and after calving in cows fed a diet restricted in calcium and phosphorus during the prepartum period. Additionally, a diet limited in calcium and phosphorus during the close‐up period has been shown to be effective in preventing clinical hypocalcemia, regardless of the number of calvings [4].

It has also been reported that the use of mineral‐vitamin supplements in the form of oral boluses, with or without concurrent injectable supplementation, is useful to increase the quantity and quality of milk production as well as some colostrum components in cows [5].

Vitamin E functions as an antioxidant by scavenging oxygen free radicals within tissues and preventing lipid peroxidation. Consequently, it preserves the integrity of membrane phospholipids against oxidative damage and peroxidation [6].

Oxidative stress is occurring in hypermetabolic dairy cows during the transition time due to an increase in reactive oxygen species (ROS) and/or impairment of antioxidant capacity [7].

There are limited studies evaluating the combination of subcutaneous AD3EC vitamins and Ca‐P‐Mg supplementation in postpartum cows. Also, maintaining cattle health during various management stages, especially the postpartum period, is important, because cows are more susceptible to various diseases during this period. The enzymatic and nonenzymatic antioxidants have a crucial role in animal health.

According to the above, the present study aimed to evaluate the effect of subcutaneous administration of vitamin (AD3EC) and mineral (calcium, phosphorus, and magnesium) supplements in dairy cows with the same calving number on serum oxidative stress parameters as a method of supplement administration in industrial management, compared to the routine method that uses an oral calcium bolus.

## 2. Methods

### 2.1. Animals and Experimental Design

Based on the sample size determination performed prior to the onset of the study, 30 apparently healthy Holstein dairy cows were selected. All cows were fed an identical diet but received different vitamin and mineral supplements during the dry period and immediately postpartum.

Group A (*n* = 15): Received a subcutaneous injection of a vitamin supplement (AD3EC) on Day 210 of gestation (single dose) and a single subcutaneous dose of calcium, phosphorus, and magnesium immediately after calving (As a method to enhance the antioxidant immune system of dairy cows due to antioxidant effects of vitamins and minerals, particularly magnesium).

Group B (control group) (*n* = 15): Did not receive any vitamin supplementation during the dry period but received an oral calcium bolus immediately after calving.

Each group was further divided into subgroups based on parity (first, third, and fifth parity): A1, A2, A3 and B1, B2, B3, respectively.

### 2.2. Sample Collection and Preparation

On Day 30 postpartum, blood samples were collected from all cows via the coccygeal vein under sterile conditions. Blood samples were transported to the laboratory on ice. Serum was separated by centrifuging the samples at 2500 rpm for 15 min.

### 2.3. Biochemical Analyses

Serum levels of total antioxidant capacity (TAC), GPX, SOD, catalase, MDA, and vitamin D were measured. Commercial kits from ZellBio GmbH, Germany, were used for TAC, GPX, SOD, catalase, and MDA, following the colorimetric method, and analyzed with an ELISA reader. Serum vitamin D levels were assessed using laboratory kits according to standard protocols.

The TAC of the samples was measured using colorimetric reactions. In this method, a buffer was combined with chromogen powder to prepare a chromogenic solution. The chromogen acted as a chemical agent, inducing color changes proportional to the TAC level in the sample due to antioxidant reactions. The absorbance intensity was measured at a wavelength of 460–490 nm using an ELISA reader.

The measurement principle of GPX is based on the enzyme utilizing GSH as the final electron donor to regenerate the reduced form of selenocysteine. The activity of GPX is inversely correlated with color formation. In this process, reduced GSH is converted to oxidized glutathione (GSSG) by the action of the GPX enzyme. After completion of the reactions, the photometric results of the assay were obtained using an ELISA reader at a wavelength of 420 nm. Subsequently, the results were calculated using the following formulas [8] (OD is an abbreviation of optical density), and the activity of GPX was determined:

(1) GP×activityU/mL=OD Control–OD SampleOD Standard–OD Blank×6000.

In the SOD assay kit, superoxide anions were converted into hydrogen peroxide and oxygen under enzymatic reaction conditions. The final product was converted into a chromogen, and the intensity of the resulting color, which is directly proportional to the enzyme activity in the samples, was measured colorimetrically at a wavelength of 420 nm using an ELISA reader. Subsequently, the activity of SOD was calculated using the following formula [8]:

(2) SOD activityU/mL=Vp–VcVp×100,

(3) Vp=OD Sample 22min–OD blank min,

(4) Vc=OD Sample 00min–OD blank min.

In the catalase assay, the decomposition of hydrogen peroxide into water and oxygen initially occurred under the action of the catalase enzyme. The rate of hydrogen peroxide decomposition is proportional to the concentration of catalase in the sample. Subsequently, the remaining hydrogen peroxide reacted with a chromogen, and the resulting color change was measured using an ELISA reader. Finally, the catalase activity was calculated using the following formula [9]:

(5) Catalase activityU/mL=OD blank–OD Sample×271×160×Sample Volume.

In the measurement of MDA, the reaction between MDA and thiobarbituric acid (TBA) under high‐temperature conditions was utilized to form an MDA–TBA complex. This reaction was carried out in an acidic environment at a temperature of 90°C–100°C, and the resulting pink‐colored product was measured at a wavelength of 535 nm.

Subsequently, a standard curve was plotted based on the optical density readings, and the concentration of MDA was determined accordingly.

The principle of this immunoenzymatic method is based on a competitive assay. In this technique, after extraction, the vitamin D present in the samples competes with enzyme‐conjugated 25‐OH vitamin D for binding to monoclonal anti‐25‐OH vitamin D antibodies coated on the wells. There is an inverse relationship between the amount of 25‐OH vitamin D‐HRP bound to the monoclonal antibody and the concentration of 25‐OH vitamin D in the sample.

Following incubation and removal of unbound analytes, the substrate tetramethylbenzidine (TMB) is added, which reacts with the enzyme to produce a blue color. The addition of a stop solution converts the blue color to yellow. The intensity of the resulting color, which is inversely related to the concentration of 25‐OH vitamin D in the sample, is measured at a wavelength of 450 nm with a reference at 630 nm.

The concentration of 25‐OH vitamin D in the samples is determined using a standard curve generated by the ELISA reader. In this curve, the absorbance values of the standards are plotted on the vertical axis at 450 nm (with a reference at 630 nm), and their concentrations (expressed in ng/mL) are plotted on the horizontal axis. Using this standard curve and the absorbance values obtained from the samples, the concentration of 25‐OH vitamin D is calculated.

### 2.4. Data Analysis

Statistical analysis was performed using SPSS Software Version 27. The normality of data distribution was evaluated using the nonparametric Shapiro–Wilk test. Most groups exhibited a normal distribution (*p* > 0.05). Therefore, the nonparametric Mann–Whitney test was used to compare means between groups for these variables.

## 3. Results

**FIGURE 1:**
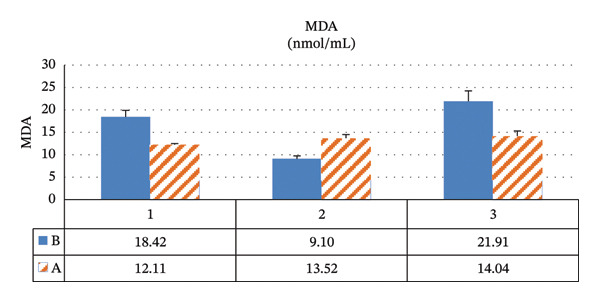
Bar chart of the mean serum malondialdehyde (MDA) levels in the study groups.

**FIGURE 2:**
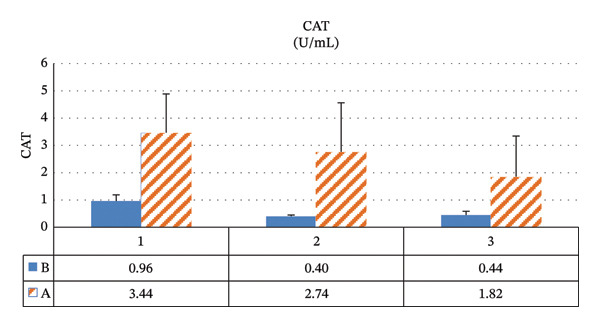
Bar chart of the mean serum catalase levels in the study groups.

**FIGURE 3:**
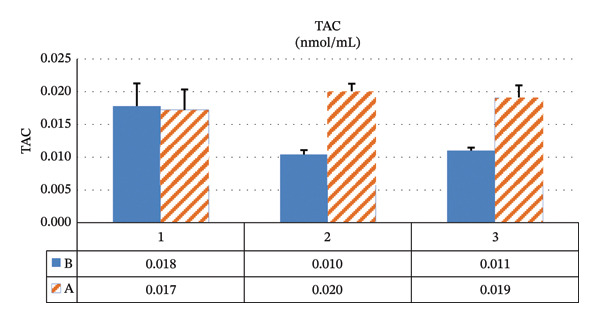
Bar chart of the mean total antioxidant capacity (TAC) levels in the serum of the study groups.

**FIGURE 4:**
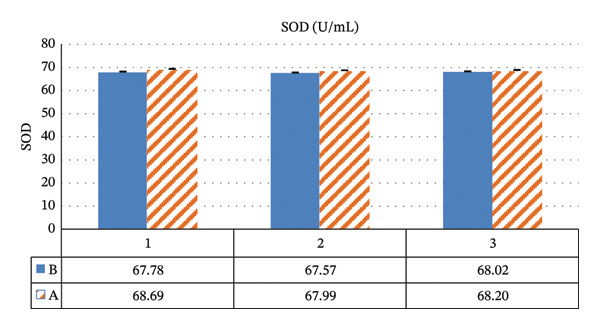
Bar chart of the mean superoxide dismutase (SOD) levels in the serum of the study groups.

**FIGURE 5:**
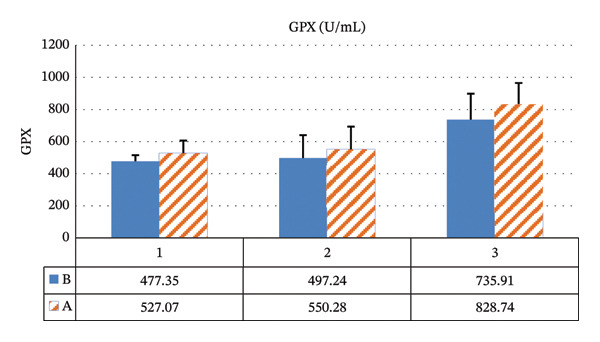
Bar chart of the mean glutathione peroxidase (GPx) levels in the serum of the study groups.

**FIGURE 6:**
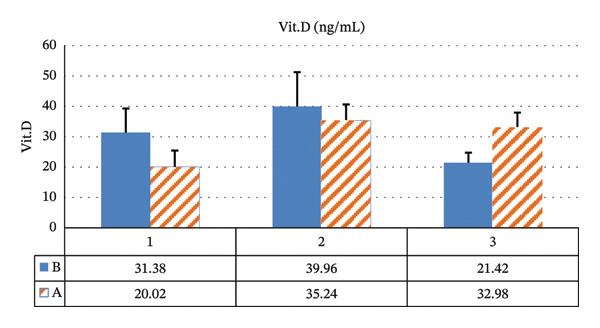
Bar chart of the mean vitamin D levels in the serum of the study groups.

The findings of the present study are presented in Table 1 and Figures 1, 2, 3, 4, 5, and 6.

**TABLE 1:** Mean ± standard deviation of the studied serum parameters in different groups.

Parameters groups	MDA (nmol/mL)	CAT (U/mL)	TAC (nmol/mL)	SOD (U/mL)	GPX (U/mL)	Vit D (ng/mL)
A1	16.06 ± 0.38^∗^	3.442 ± 1.44	0.017 ± 0.003	68.69 ± 0.38	527.07 ± 78.86	20.02 ± 5.42
A2	13.52 ± 4.26^∗∗^	2.738 ± 1.82^∗∗^	0.020 ± 0.01^∗∗^	67.99 ± 1.0	550.28 ± 142.43	35.24 ± 5.38
A3	14.04 ± 1.26^∗∗∗^	1.818 ± 1.52	0.019 ± 0.002^∗∗∗^	68.20 ± 0.67	828.74 ± 135.77	32.98 ± 4.95
B1	18.42 ± 1.47	0.956 ± 0.23	0.018 ± 0.0035	67.78 ± 0.34	477.35 ± 37.58	31.38 ± 7.91
B2	9.10 ± 0.66^#^	0.400 ± 0.05	0.010 ± 0.0007	67.57 ± 0.17	497.24 ± 142.29	39.96 ± 11.31
B3	21.91 ± 2.33	0.444 ± 0.14	0.011 ± 0.0005	68.02 ± 0.20	735.91 ± 162.1	21.42 ± 3.32

^#^Indicates a significant difference between group B2 and groups B1 and B3.

^∗^Indicates a significant difference between group A1 and B1.

^∗∗^Indicates a significant difference between group A2 and B2.

^∗∗∗^Indicates a significant difference between group A3 and B3.

Analysis of the tables and figures indicated a significant difference in mean MDA levels only between subgroups B2 and B1 (*p* = 0.008) (Table 1) when comparing the mean levels of each parameter within groups A and B.

Comparing mean values of each parameter between corresponding subgroups of groups A and B revealed significant differences in mean MDA levels. Specifically, mean MDA levels in subgroups A1 and A3 were significantly lower than in subgroups B1 and B3, respectively, while the mean MDA level in subgroup A2 was significantly higher than in subgroup B2.

Regarding mean catalase levels, statistical analysis revealed a significant difference between groups A and B (*p* = 0.008), which was attributed to the elevated catalase level observed in subgroup A2 compared to subgroup B2 (Table 1).

Additionally, a significant difference was observed between the mean TAC levels of subgroups A2 and B2, as well as A3 and B3. This difference is attributed to the elevated TAC levels in the subgroups of group A (*p* = 0.008) (Table 1).

No significant differences were observed between any of the other studied parameters in groups A and B (*p* ≥ 0.05).

## 4. Discussion

Lipid peroxidation can activate the inflammatory process. The activation of this process not only alters nutrient metabolism but also contributes to increased production of free radicals and lipid peroxidation. Additionally, free radicals may affect immune cells similarly to other body cells, leading to their destruction and subsequently reducing the animal’s resistance to pathogenic agents [10].

Immune cells are highly sensitive to oxidative stress due to the abundance of unsaturated fatty acids in their membranes [10]. Stimulation of these cells can lead to the production of large amounts of ROS.

As described in the methodology, this study examined two groups of Holstein dairy cows to evaluate the effect of vitamin and mineral supplementation on oxidative stress status.

In this study, sampling was performed on Day 30 postpartum to allow the used supplements sufficient time to affect the body’s antioxidant immune system. In this time reproductive system inflammation and infections caused by childbirth are expected to be minimized. Therefore, the effect of the supplements on the immune system can be better investigated at this time (when the final clean test is performed).

As observed in the table, within each of the main study groups (A and B), there was no significant difference in the mean levels of any measured parameters among the internal subgroups (*p* ≥ 0.05). The only significant difference was in the mean MDA level between subgroup B2 and subgroups B1 and B3.

It appears that in the group that received oral calcium tablets immediately after calving without vitamin and mineral supplementation, the MDA level was considerably higher in the first and fifth parities compared to the third parity. However, in the group that received vitamin and mineral supplementation, no significant difference in MDA levels was observed among the subgroups.

A significant difference exists in the mean serum MDA levels between groups A1 and B1, A2 and B2, and A3 and B3. This difference is attributed to the lower MDA levels in the A subgroups. In other words, the single‐dose subcutaneous administration of the vitamin‐mineral supplement led to a reduction in MDA production. Given the antioxidant properties of minerals like copper and zinc, as well as various vitamins, it is expected that supplementation containing these compounds could neutralize the oxidative agents responsible for lipid peroxidation and free radical production, thereby preventing lipid peroxidation and subsequent MDA formation.

Although the administration of the vitamin‐mineral supplement in Group A significantly increased catalase levels in subgroup A2 compared to B2, as well as TAC in subgroups A2 and A3 compared to B2 and B3. It did not have a significant effect on catalase levels in other groups or on the levels of SOD, GPX, and vitamin D.

Considering the reduction in MDA levels across all A subgroups compared to their corresponding B subgroups and the significant increase in TAC levels in subgroups A2 and A3, it should be noted that other antioxidants studied were not substantially affected by the single‐dose subcutaneous administration of the vitamin‐mineral supplement.

However, the administration of this supplement appears to have influenced other antioxidants not measured in this study.

The considerable increase in catalase levels in third‐parity cows that received a single subcutaneous dose of the vitamin‐mineral supplement, along with the absence of a significant increase in catalase levels in groups A1 and A3 compared to groups B1 and B3, suggests that both repeated supplementation and the cow’s parity may play a role in catalase production.

The significantly lower MDA levels in third‐parity cows that did not receive the vitamin‐mineral supplement, compared to first‐ and fifth‐parity cows in the same group, suggest that the antioxidant system is more active and oxidative stress is lower in third‐parity cows. Consequently, the significant increase in catalase levels in third‐parity cows receiving the vitamin‐mineral supplement, compared to first‐ and fifth‐parity cows in the same group, is likely more related to the effect of age rather than repeated administration of the supplement due to a higher number of parities.

In this regard, Kayar et al. (2025) proved supplementing the ration with a premix containing antioxidant substances during the transition period can be beneficial for terms of maintaining the body condition score (BCS) balance, animal welfare, and herd health [11].

Saeedian et al. (2025) observed that AD3EC vitamin can reduce oxidative stress and promote overall health and resilience in Raini cashmere goats during the transition period [12].

Sharma et al. (2011) explained dairy cows have more oxidative stress and low antioxidant defense during early lactation or just after parturition than advanced pregnant cows which is probably the reason for their increased susceptibility to production diseases [13].

Khodamoradi et al. (2019) reported that separate administration of copper and vitamin C induces changes in the count of certain blood cells in cows. But the simultaneous injection of vitamin C and copper in transition cows under heat stress does not significantly affect milk production and composition during the first 90 days of lactation [14].

Regarding the effect of mineral supplements on increasing mineral and vitamin levels, Hossein Pour et al. (2010) stated that including zinc supplements in the diet of dairy cows increases serum zinc levels, thereby improving metabolism and immune function. Also elevates serum vitamin A levels, preventing the adverse effects of vitamin A deficiency. However, they reported no significant effect on β‐carotene levels. These researchers concluded that supplementing zinc in the diet of dairy cows is essential [15].

Moshref et al. (2015) determined in their study, compared the jugular vein, mammary vein, and coccygeal vein, that the coccygeal vein is the most suitable vessel for blood sampling to assess oxidative status. They also reported that MDA levels and antioxidant concentrations vary among different blood vessels [16].

Concerning the effect of parity on blood calcium and phosphorus levels, Faramarzyan et al. (2016) reported that the number of calvings does not significantly affect the incidence of subclinical hypocalcemia before and after parturition in cows fed a diet with restricted calcium and phosphorus during the close‐up period. They concluded that this diet is effective in preventing clinical hypocalcemia, regardless of parity [4].

Mousavi et al. (2020) reported that prepartum injection of vitamin B12, vitamin E, and the trace elements iron and selenium had no effect on the birth weight of calves or the percentage of fat, protein, lactose, solids‐not‐fat, and IgG concentration in colostrum. Additionally, these minerals and vitamins did not influence the activity levels of GPX, CAT, TAC, or serum metabolite concentrations in calves. The researchers concluded that administering this supplement at 21 and 7 days prepartum had no impact on the immune system of calves within the first 24 h of life [3].

Akin et al. (2013) stated that injection of 10 mg of vitamin B12 solution from 60 days before to 150 days after parturition in dairy cows had no effect on colostrum protein percentage or IgG concentration [17].

Numerous studies have shown that plasma vitamin E levels in dairy cows decrease near parturition due to impaired vitamin E transport in plasma and increased fat accumulation in the liver [18, 19]. Therefore, researchers have considered vitamin E injection during the transition period, particularly close to parturition, to be necessary [20, 21].

Zhang et al. (2024) reported INC supplementation consumption in the transition period has anti‐inflammatory and antioxidative stress effects. Also, it can improve the performance of dairy cows [22].

It is reported that use of antioxidants supplementation before calving caused increased insulin sensitivity after calving; it can be due to the potential benefits of antioxidant supplementation in decreasing the consequences of negative energy balance [23].

## 5. Conclusion

In the present study, the consumption of mineral (calcium, phosphorus, and magnesium) and vitamin (AD3EC) supplements does not significantly alter the mean concentrations of SOD, GPX, and vitamin D. However, a significant decrease in MDA and an increase in TAC and catalase levels were observed. So it can be concluded that the consumption of mineral and vitamin supplements reduces oxidative stress by improving some oxidative parameters in Holstein dairy cows.

Future studies are needed to investigate the long‐term effects of vitamin and mineral supplementation on dairy cow health and production.

**Nomenclature**
MDA: Malondialdehyde
TAC: Total antioxidant capacity
SOD: Superoxide dismutase
GPX: Glutathione peroxidase
CAT: Catalase

## Funding

No funding was received for this manuscript.

## Ethics Statement

The study received ethical approval whit approval no: IR.IAU.KAU.REC.1403.051.

## Conflicts of Interest

The authors declare no conflicts of interest.

## Data Availability

The data that support the findings of this study are available from the corresponding author upon reasonable request.

## References

[1] Yang F. L. , Li X. S. , and He B. X. , Effects of Vitamins and Trac_Elements Supplementation on Milk Production in Dairy.Cows: A Review, African Biotechnology Journal. (2011) 10, 2574–2578, 10.5897/AJB10.2025.

[2] Karkoodi K. , Chamani M. , Beheshti M. , Mirghaffari S. S. , and Azarfar A. , Effect of Organic Zine Maganese, Copper and Selenium Chelates on Colostrum Production and Reproductive and Lameness Indices in Adequately Supplemented Holstein Cow, Journal of Biology Trace Element Research. (2012) 146, no. 1, 42–46, 10.1007/s12011-011-9216-5. 21965109

[3] Musavi S. R. , Fatania F. , Tasoli G. et al., Effect of Ingection of Vitamin E and Selenium Solution and Vitamin B and Iron Solution to Transition Dairy Cows on Colostrum Quality and Antioxidant Capacity and Serum Metabolites in Calves, Iranian Journal of Animal Science. (2020) 50, no. 4, 307–317, 10.22059/IJAS.2019.269845.653670.

[4] Faramarzian K. , Magikolnei H. , Nouri M. R. et al., Evaluation of Clinical and Subclinical Hypocalcemia in Primiparous and Multiparous Coms Consumed Limited Calcium and Phosphorus Diet in Close up Period, Iranian Veterinary Journals. (2017) 13, no. 2, 58–62.

[5] Khorsandi S. , Riasi A. , Khorosh M. et al., The Effect of Some Mineral-Vitamin Supplements on the Quantity and Quality of Milk Production and Colostrum Quality of High-Yielding Holstein Cow in the Summer, Journal of Animal Science Research. (2013) 23, no. 2, 87–93.

[6] Vahidi A. and Karimi R. , Fertility in Dairy Cows,” Challenges and Solutions, 2018, University of Tehran,Publishing.

[7] Younis M. , Ei-Ashker M. , Ei-Diasty M. et al., Oxidative Stress in Transition Dairy Cattle: Current Knowledge and the Potential Impact of Supplementing Organic Trace Elements, Asian Journal of Research in Animal and Veterinary Sciences. (2020) 7, no. 1, 1–2.

[8] Jamshidi H. R. and Kalantar H. , Effect of Sodium Selenide on Renal Toxicity Induced by Mercuric Chloride in Rat, International Journal of Medical Laboratory. (2020) 7, no. 2, 90–101, 10.18502/ijml.v7i2.2912.

[9] Fatemi M. , Ghandehari F. , Fatemi E. , and Fatemi Y. , The Effect of *Lactobacillus fermentum* Against Lead-Induced Oxidative Damages in Rat Kidneys, Iranian Journal of Toxicology. (2023) 17, no. 1, 53–62, 10.32598/IJT.17.1.596.2.

[10] Spears J. W. and Weiss W. P. , Role of Antioxidants and Trace Eelements in Health and Immunity of Transition Dairy Cows, The Veterinary Journal. (2008) 176, no. 1, 70–76, 10.1016/j.tvjl.2007.12.015. 18325801

[11] Kayar T. , Ozkurt G. , Erzurum O. et al., Thiol/Disulphide Homeostasis in the Relationship Between Body Condition Score and Oxidative Stress in Periparturient Period Holstein Heifers, Veterinary Medicine and sceinces. (2025) 11, no. 3, 10.1002/vms3.70326. PMC11970299 40184051

[12] Saeedian A. , Sebdani M. M. , Shamsaddini Bafti M. , and Kargar N. , Evaluating the Efficacy of Injectable Antioxidant AD3EC on Oxidative Stress Biomarkers in Raini Cashmere Goats, BMC Veterinary Research. (2025) 21, no. 1, 10.1186/s12917-025-05091-2. PMC12629044 41257721

[13] Sharma N. , Singh N. K. , Sing O. P. , Pandey V. , and Verma P. K. , Oxidative Stress and Antioxidant Status During Transition Period in Dairy Cows, Asian–Australia Journal Animal and Sciences. (2011) 24, no. 4, 479–484, 10.5713/ajas.2011.10220.

[14] Khodamoradi S. H. , Fatahnia F. , and Jafari Taasoli G. , Effect of Injection of Vitamin C and Copper on Milk Production and Composition and Body Condition Score of Transition Dairy Cows Under Heat Stress, Journal of Ruminant Research. (2019) 3, 77–92.

[15] Hossein Pour A. , Ghasemzadeh E. , and Davoodi Y. , Investigation of the Effect of Intestinal Supplementation on Serum Zinc, Vitamin A and Beta Carotene Levels in Dairy Cows, Veterinary Journal of Islamic Azad University. (2010) 4, no. 11, 63–68.

[16] Moshref M. , Aslani M. , Mohebbi A. et al., Evaluation of the Oxidative Status of Blood Samples Taken From the Jugular, Mammary, and Subcaudal Veins in Dairy Cows, Iranian Journal of Clinical Veterinary Sciences. (2015) 9, no. 2, 63–68.

[17] Akin S. M. , Bertics S. J. , Sacha M. T. et al., Effects of Ghalt Supplementation and Vitamin B Injection’s on Lactation Performance and Metabolism of Holstein Doing Cons, Journal of Dairy Science. (2013) 96, no. 3, 1755–1768, 10.3168/jds.2012-5979. 23312998

[18] Baldi A. , Savoini depinotti L. , Montardini F. et al., Official Methods of Analytical, 2008, 17th edition, Arlington.

[19] Hogan J. S. , Weiss W. P. , and Smith K. L. , Role of Vitamin E and Selenium in Host Defense Against Mastitis, Journal of Dairy Science. (1993) 76, no. 9, 2795–280, 10.3168/jds.S0022-0302(93)77618-3. 8227683

[20] Erskine R. J. , Bartlett P. C. , and Herdt Tand daston P. , Effects of Parenteral Administration of Vitamin on Health of Prepartum Dating Course, Journal of the American Veterinary Medical Association. (1997) 211, 466–462. 9267510

[21] Pontes G. C. S. , Manteiro P. L. E. , Prata A. B. et al., Effect of Injectable Vitamin E on Incidence of Retained Fetal Membranes and Reproductive Performance of Dairy Cows, Journal of Dairy Science. (2015) 98, no. 4, 2437–2449, 10.3168/jds.2014-8886. 25682134

[22] Zhang H. , Nuermaimaiti Y. , Hao K. et al., Supplementation With Combined Additive Improved the Production of Dairy Cows and Their Offspring With Maintenance of Antioxidative Stability, Antioxidants. (2024) 13, no. 6, 10.3390/antiox13060650. PMC11200508 38929089

[23] Abuelo A. , Alves-Nores V. , Hernandez J. , Muiño R. , Benedito J. , and Castillo C. , Effect of Parenteral Antioxidant Supplementation During the Dry Period on Postpartum Glucose Tolerance in Dairy Cows, Journal of Veterinary Internal Medicine. (2016) 30, no. 3, 892–898, 10.1111/jvim.13922. 26971714 PMC4913581

